# Chang’E-5T Orbit Determination Using Onboard GPS Observations

**DOI:** 10.3390/s17061260

**Published:** 2017-06-01

**Authors:** Xing Su, Tao Geng, Wenwen Li, Qile Zhao, Xin Xie

**Affiliations:** GNSS Research Center, Wuhan University, 129 Luoyu Road, Wuhan 430079, China; suxing_gnss@whu.edu.cn (X.S.); cheeselee@whu.edu.cn (W.L.); zhaoql@whu.edu.cn (Q.Z.); xiexin@whu.edu.cn (X.X.)

**Keywords:** Chang’E-5T, orbit determination, onboard GNSS receiver, C/N0, deep space navigation

## Abstract

In recent years, Global Navigation Satellite System (GNSS) has played an important role in Space Service Volume, the region enclosing the altitudes above 3000 km up to 36,000 km. As an in-flight test for the feasibility as well as for the performance of GNSS-based satellite orbit determination (OD), the Chinese experimental lunar mission Chang’E-5T had been equipped with an onboard high-sensitivity GNSS receiver with GPS and GLONASS tracking capability. In this contribution, the 2-h onboard GPS data are evaluated in terms of tracking performance as well as observation quality. It is indicated that the onboard receiver can track 7–8 GPS satellites per epoch on average and the ratio of carrier to noise spectral density (C/N0) values are higher than 28 dB-Hz for 90% of all the observables. The C1 code errors are generally about 4.15 m but can be better than 2 m with C/N0 values over 36 dB-Hz. GPS-based Chang’E-5T OD is performed and the Helmert variance component estimation method is investigated to determine the weights of code and carrier phase observations. The results reveal that the orbit consistency is about 20 m. OD is furthermore analyzed with GPS data screened out according to different C/N0 thresholds. It is indicated that for the Chang’E-5T, the precision of OD is dominated by the number of observed satellite. Although increased C/N0 thresholds can improve the overall data quality, the available number of GPS observations is greatly reduced and the resulting orbit solution is poor.

## 1. Introduction

GNSS has evolved into a more robust system in recent years and played a vital role in scientific and engineering applications. It was originally conceived to provide the terrestrial and airborne users with positioning, navigation, and timing services. Later, the services were adopted in low Earth orbit (LEO) satellites applications, such as real-time spacecraft navigation, precise orbit determination, three-axis attitude control, precise time synchronization, etc. [1,2,3,4]. The performance environment of GNSS receiver in LEO is similar to the terrestrial applications, apart from the highly dynamic effects due to orbital velocity. Based on the GPS measurements for LEO, the accuracy of LEO orbit determination has been improved to the centimeter level [5,6]. The service region that encloses the terrestrial users to all users up to 3000 km with similar service performance is termed as Terrestrial Service Volume (TSV) [7].

The region spanning the altitudes above 3000 km up to 36,000 km is considered as the Space Service Volume (SSV) [8,9]. This can be further divided into two regions: (1) the medium Earth orbit (MEO) SSV (3000~8000 km); (2) the high Earth orbit (HEO)/geostationary Earth orbit (GEO) SSV (8000~36,000 km). There is a widespread interest in the extension of GPS-based navigation to SSV users, because it could maximize the autonomy of a spacecraft and reduce the burden and costs of ground infrastructure operations. However, the use of GPS signals for SSV users, particularly for HEO/GEO missions, has special design challenges. In these missions, the GNSS receiver is at an altitude above the altitude of the GPS constellations. Consequently, the onboard GNSS receivers could only capture the side-lobe signals or main-beam signals originated from satellites on the opposite side of the Earth [10,11,12]. The power of side-lobe signals are generally about 20 dB lower than that of the main-beam ones, which would lead to the poorer observation quality [10]. The main-beam signals are 10 to 100 times weaker with limited satellite spatial diversity [13]. Moreover, the GPS satellites at high altitude, depending on the receiver sensitivity, can drastically reduce the navigation solution accuracy of SSV users, due to the very limited number of visible satellites and very poor tracking geometry for the region in the field of view [14,15,16]. In spite of these difficulties, specialized GNSS receivers have been designed with increased signal acquisition and weak signal tracking capabilities because of receiver technology improvements and navigation signal modernization [17], such as the Navigator GPS receiver developed at the NASA Goddard Space Flight Center [18], the TOPSTAR 3000 receiver of the European Space Agency (ESA) [19], and the low-cost GNSS system funded by the Italian Space Agency [20]. The concept of GPS tracking in SSV has actually been demonstrated by some flight experiments. For example, the EQUATOR-S spacecraft tracked a GPS satellite from an altitude of about 61,000 km and demonstrated the possibility of a reception of GPS side-lobe signals within a very high eccentricity orbit [21]. A GPS receiver flying on the AMSAT-OSCAR 40 (AO-40) spacecraft at a high inclination, 1000 km by 58,800 km altitude orbit, was developed to support the use of GPS for HEO experiments [22,23]. The reference orbit generated from the Two-Line Ephemeris (TLE) and the Simplified Deep-space Perturbation Version 4 (SDP4) propagator showed to be in error by about 100 km [24]. In addition, the possibility and feasibility to exploit GNSS navigation in lunar trajectories were also investigated and analyzed [25,26]. 

Chang’E 5T was launched by China National Space Administration on 24 October 2014. The mission of Chang’E 5T is lunar flyby and Earth re-entry to conduct crucial tests on capsule design planned to be used in the Chang’E-5 mission. The Chang’E-5T lunar probe is equipped with a high sensitivity GNSS receiver which could capture the GPS and GLONASS code and carrier wave signal. Fang et al. [27] firstly proved the idea of using rocket GPS measurements to determine the injected transfer orbit of Chang’E-5T. Fan et al. [28] conducted Chang’E-5T OD using onboard GPS pseudo-range measurements by dynamic method, and results showed that the position error in the one-h prediction from the OD of 1.5 h arc is less than 109 m. However, the higher accuracy carrier phase measurements were not used in Fang et al. [27] or Fan et al. [28]. For the combined use of pseudo-range and carrier phase measurements in SSV, weighting approach needs to be investigated because of the lack of a priori measurement precision information compared to TSV users. In addition, the specially designed receiver was used in SSV. As an important indicator of GNSS receiver design, the C/N0 given by a receiver would affect the number of visible satellite and the quality of GNSS measurements [29], which is crucial for the OD precision. 

Consequently, the GPS tracking data quality analysis and GPS-based orbit determination results for Chang’E-5T, as well as their relationship with the C/N0 are discussed in this contribution. The paper is organized as follows: In Section 2, we analyze Chang’E-5T onboard GPS data sensitivity and availability. The number of visual GPS satellites, position dilution of precision (PDOP) values and C1 code errors as a function of time and C/N0 value during the tracking arc are discussed. In Section 3, the Chang’E-5T orbit is determined using un-differenced combined observations of L1 carrier phase and the C1 code. The orbit determination period is 19:20–21:20 31 October 2014 and split into two arcs (19:20–20:50, 19:50–21:20) for the overlap arc to test the orbit consistency. Comparison between dynamic and kinematic orbit solutions is analyzed. Finally, the OD precisions with different C/N0 thresholds are discussed.

## 2. Chang’E-5T Onboard GPS Data Analysis

Chang’E-5T lunar probe was launched at 18:00 UTC, 23 October 2014 from Xichang Satellite Launch Center of China and entered the orbit from Earth to Moon with the perigee altitude 209 km and apogee altitude 413,000 km. After the 196 h Earth-Moon-Earth flight, the probe successfully returned to Earth. The service module and the return vehicle were separated on 21:53 UTC, 31 October 2014 at the altitude of about 5000 km [30].

To examine the sensitivity of navigation performance during the mission of lunar exploration and availability of GNSS and to target a precise re-entry corridor in the Earth’s atmosphere, a GNSS receiver capable of acquiring and tracking weak signals with high sensitivity was installed in the spacecraft. According to the flight operation schedule of Chang’E-5T mission, the GNSS receiver worked only twice, 18:56–21:53, 23 October and 18:55–21:56, 31 October. The task of Arc 1 was to demonstrate the reception of GNSS signals and to verify whether the receiver worked normally. The objective of Arc 2 was to evaluate the prediction accuracy of the separation point between the service module and the return vehicle. Unfortunately, there was a data slip in the middle of Arc 1 that lasted approximately 3 min [28]. As a result, the Arc 2 is chosen to be analyzed here.

The GNSS receiver could omni-directionally track GPS and GLONASS L1 phase and code observations with two antennae mounted on opposite sides of spacecraft, one of which is Earth-pointing direction and the other oriented in the zenith direction. This receiver is capable of tracking up to 24 satellites simultaneously with 24 channels. Since the GLONASS tracking data are much less and the accuracies are poorer than that of GPS [28], we didn’t introduce the contribution of GLONASS in Chang’E-5T OD. The data collected by the receiver include the carrier phase, pseudo-range, Doppler and C/N0 measurement of L1 band in Receiver Independent Exchange Format (RINEX) 2.0 format. Table 1 provides some information about this receiver.

### 2.1. Onboard GPS Data

**Figure 1:**
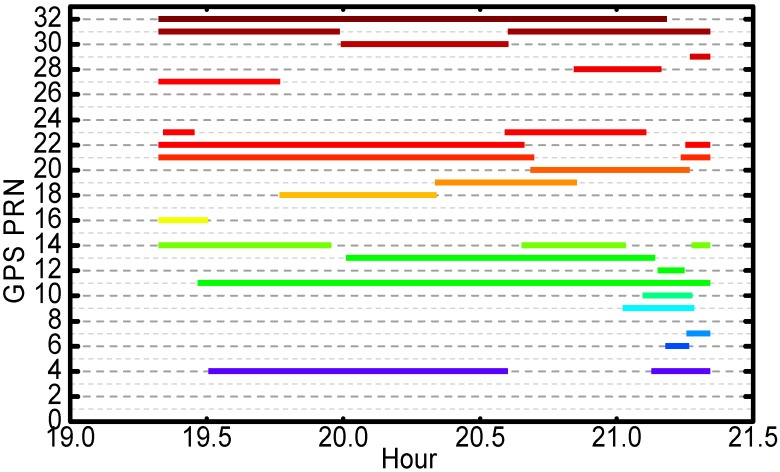
GPS satellites tracked by Chang’E-5T onboard receiver on 31 Octomber 2014.

As shown in Figure 1, during the return periods, the receiver was able to acquire and track at least 22 GPS satellites. In most of time, the signals from eight GPS satellites were tracked simultaneously. The length of continuous tracking periods of four GPS satellites are near two hs, for instance Pseudo Random Noise (PRN) G11, G21, G22 and G32.

**Figure 2:**
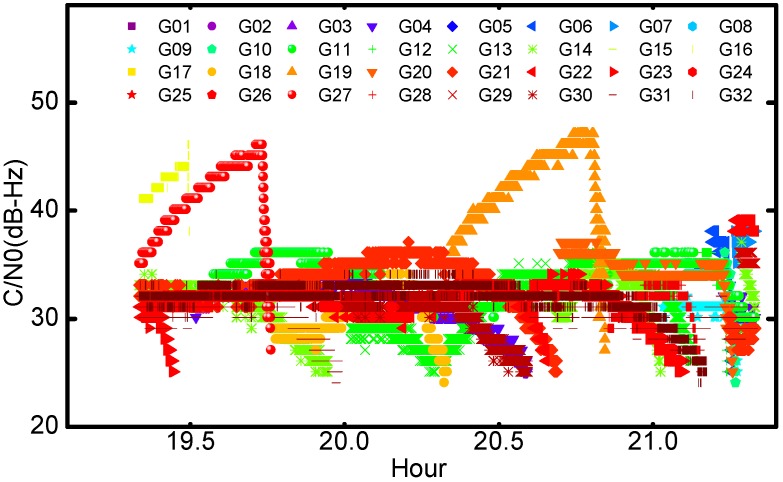
The measured signal level of GPS Satellites: C/N0 (unit: dB-Hz).

**Figure 3:**
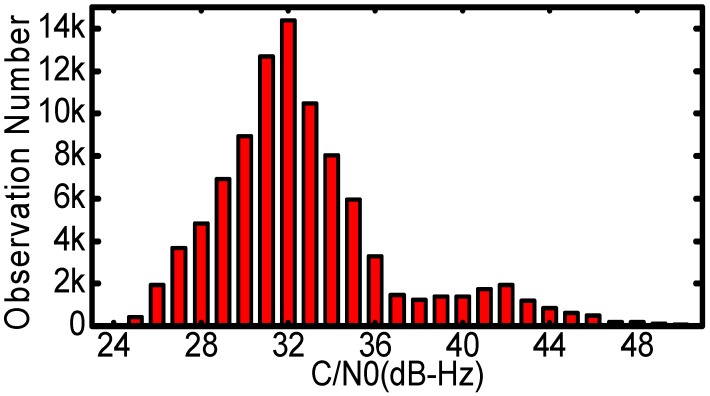
The number of observations with different C/N0 (unit: dB-Hz).

Figure 2 shows the measured signal levels of all the GPS satellites during the returning arc. It should be noted that the original C/N0 values were recorded in integers. The variations of C/N0 are very significant, approximately 25 dB-Hz peak-to-peak. For PRN G16, G19 and G27, the C/N0 values were basically larger than 35 dB-Hz because the Chang’E-5T probe was in the coverage of their main-beam signals; while the line-of-sight of the probe pierced the ionosphere and troposphere, i.e., GPS occultation, the C/N0 values dropped dramatically. Figure 3 is a statistic histogram of all the C/N0. The C/N0 values are mainly concentrated between 28 dB-Hz and 35 dB-Hz, much smaller than those of terrestrial and low earth orbit receivers. Even though the designed tracking sensitivity threshold of the GNSS receiver is 26 dB-Hz, there are also about four hundred observations weaker than the thresholds.

### 2.2. Number of Visual GPS Satellites and PDOP

**Figure 4:**
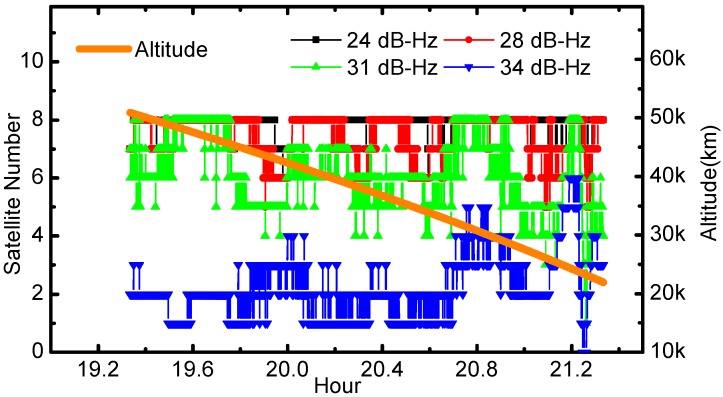
The number of observed GPS satellites with respect to C/N0. The orange line represents the corresponding altitudes of the flight arc, which are the distances from Chang’E-5T to the Earth center.

The C/N0, an important indicator of GNSS receiver design, would affect the number of visible satellite. Figure 4 shows the number of visible GPS satellites with respect to spacecraft altitude and different C/N0 thresholds, and only the GPS observations with C/N0 values larger than the threshold are utilized in calculations. The C/N0 thresholds are set as 24, 28, 31 and 34 dB-Hz, respectively. During the returning experiment arc, the altitude fell from 50,000 km to 20,000 km. The average number of tracked GPS satellites is 7.8 when the threshold of 24 dB-Hz is taken into account. Increasing the signal tracking threshold to 31 dB-Hz, the average number decreased to 6.1. Finally, an increase of the threshold from 31 dB-Hz to 34 dB-Hz witnessed a significant decrease of the average number of visible GPS satellites to 2.2.

**Figure 5:**
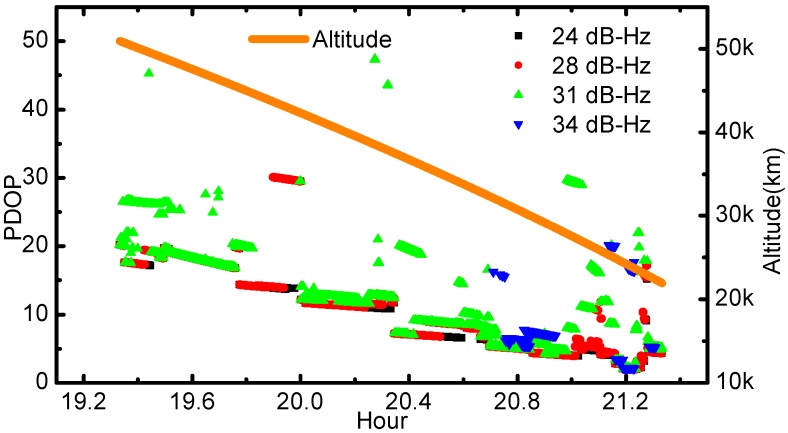
PDOP values time series according to the different C/N0 thresholds. The orange line represents the altitudes of the Chang’E-5T with respect to the Earth center.

PDOP reveals the geometry strength between the receiver and GNSS transmitters. Usually, small PDOP value indicates good geometry condition. The PDOP variations against different C/N0 thresholds are also investigated and shown in Figure 5. Since the PDOP could only be derived with simultaneously tracked signals from at least four GPS satellites, the threshold of 34 dB-Hz led to quite few results, most of which were near the earth. When the C/N0 thresholds are set as 31 dB-Hz and 34 dB-Hz, the PDOP values can even reach over 50 in some epochs and thus are not displayed in the plot. For the case of Chang’E-5T, the PDOP values are greatly affected by the altitudes. The main reason is that GPS satellites can be observed with better spatial distribution from the receiver antenna when the spacecraft is in the lower altitudes, indicating better geometry condition. 

### 2.3. C1 Code Errors

For onboard GPS signals of Chang’E-5T, most of them are ionospheric-free and tropospheric-free. It should be mentioned that only 0.2% of all the signals are affected by the atmosphere which originated from GPS satellites on the opposite part of the Earth, and excluded for computing code multipath and noise. Hence the differences between the L1 and C1 measurements used in this section include only L1 ambiguities, L1 multipath errors and noise, as well as C1 multipath errors and noise. After removing the constant ambiguities by subtracting the mean value of the differences between the L1 and C1 measurements, the residual series are dominated by the code multipath and noises since the carrier phase multipath and carrier phase noises are much smaller in magnitude. In the following the residual series is referred as C1 code errors and is used to assess the precision of C1 code observations.

**Figure 6:**
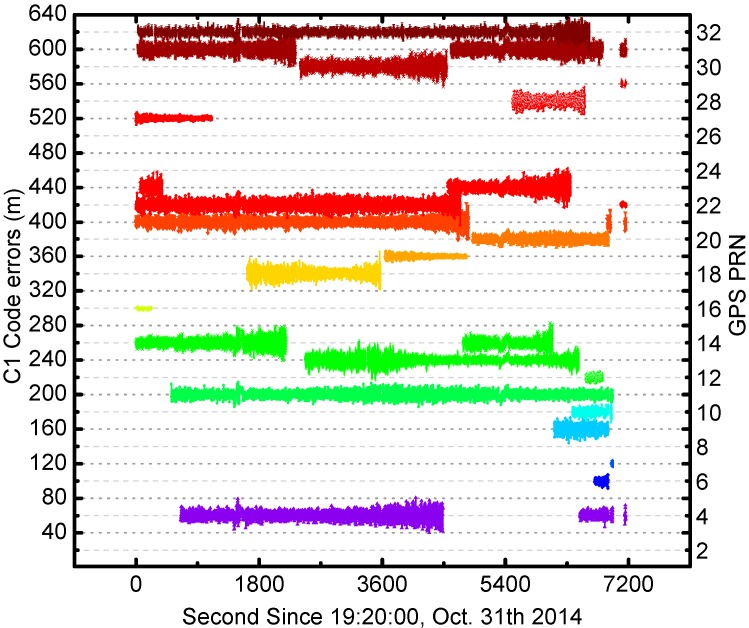
The times series of C1 code errors for all GPS satellites (unit: meter). The values of each satellite have been offset 20 m for clarity. The different colors represent different satellites, and the colors are the same as Figure 1.

**Figure 7:**
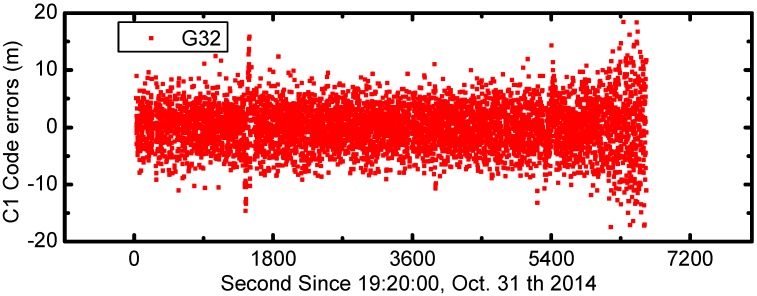
The times series of C1 code errors for G32 satellite (unit: meter).

The C1 code errors for all GPS satellites are calculated and shown in Figure 6. In the figure the C1 code errors are shifted by 20 m from one to another. The code errors are mainly between ±10 m and show consistent variations during the entire 2 h. In order to show more details of onboard GPS code measurement errors, we also plot the times series of C1 code errors for G32 satellite in Figure 7. The mean value and standard deviation of G32 code errors are 0 m and 3.92 m, respectively.

**Figure 8:**
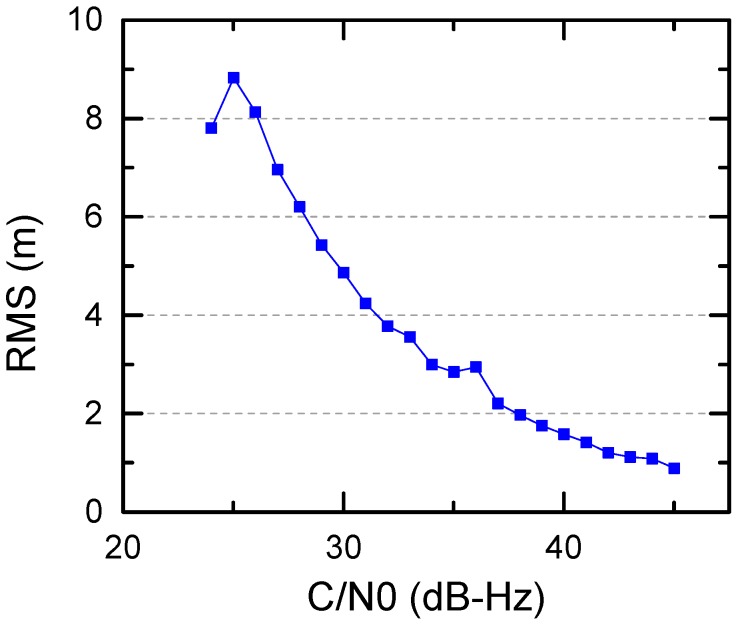
C1 code errors as a function of C/N0.

In addition, to analyze the relationship between code errors and C/N0 values, the Root Mean Squares (RMS) values of C1 code errors as a function of C/N0 are shown in Figure 8. We can see that the precision of C1 observations is better with C/N0 values increasing. The C1 code errors reach to as large as 10 m when C/N0 gets close to the lower limit of the receiver sensitivity. For C/N0 larger than 30 dB-Hz, the C1 observation errors are better than 5 m. The overall precision of C1 code errors is 4.15 m.

## 3. Chang’E-5T Orbit Determination

In this section, the orbit determination strategy is presented in detail, and the orbit estimates of Chang’E-5T are evaluated.

### 3.1. Processing Strategy

The modified version of Position and Navigation Data Analyst (PANDA) software package [31] developed by GNSS Research Center of Wuhan University is employed to conduct Chang’E-5T orbit determination in this study. Table 2 summarized the observation models, dynamical models and estimated parameters. The entire 2-h GPS L1 and C1 data, sampled at 3 s, starting from 19:20:00, 31 October 2014 is processed in a batch mode. It is noted that the Helmert variance component estimation method [32] is employed to determine the weights of code and carrier phase observations. The International GNSS Service (IGS) final orbit and 30 s clock offsets products are employed to bring high-precision coordinate and time frame. The phase-windup errors and relativistic effects are corrected using theoretical equations or empirical models. The igs08.atx antenna calibrations are used for the GPS satellites phase center offset (PCO) and phase center variation (PCV) corrections [33]. The receiver PCO and PCV are not considered as they are not available. The non-spherical gravity perturbations are computed by EIGEN_GL04C gravity model with degree and order of 50. The JPL DE405 ephemeris is used to calculate the N-body perturbations, while the solar radiation pressure is calculated using the Extended CODE Orbit Model (ECOM). The Chang’E-5T initial state vector, dynamic parameters, receiver clock errors as well as ambiguities are estimated during OD. The corresponding orbit solutions are called dynamic orbits.

The initial orbits of Chang’E-5T are obtained by Standard Point Positioning (SPP) using C1 code observations. The polynomial fitting method is used to detect outliers and cycle slips in L1 carrier-phase as well as C1 code measurements [34]. The post-fit residuals are also analyzed to detect minor cycle slips.

### 3.2. Orbit Determination

#### 3.2.1. Residual Analysis

**Figure 9:**
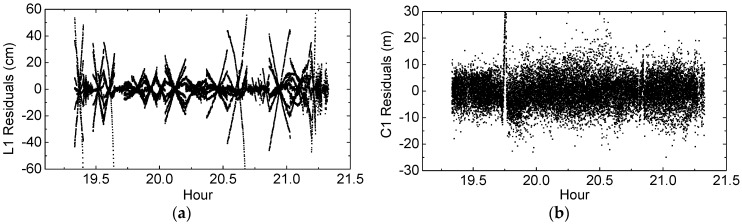
(**a**) L1 observation residual series (unit: centimeter); (**b**) C1 observation residual series (unit: meter).

For the entire 2-h arc OD results, the post-fit L1 and C1 residuals are firstly analyzed to evaluate the quality of estimated Chang’E-5T orbits. As seen in Table 2, most of the observation errors have been corrected during the OD process. Hence, large post-fit residuals often reflect poor observation modeling as well as poor orbit estimation. The L1 and C1 residuals are shown in Figure 9a,b respectively. The L1 residuals from several satellites show significant linear variations with respect to epochs, and can reach 60 cm, which may be due to carrier phase noise variations as a function of C/N0 [29] or imperfect observation models. The RMS errors of the post-fit L1 and C1 residuals are 74 mm and 4.6 m respectively, and the RMS of C1 residuals is consistent with that of C1 code errors presented in Section 2.3.

#### 3.2.2. Orbit Overlap Comparison

**Figure 10:**
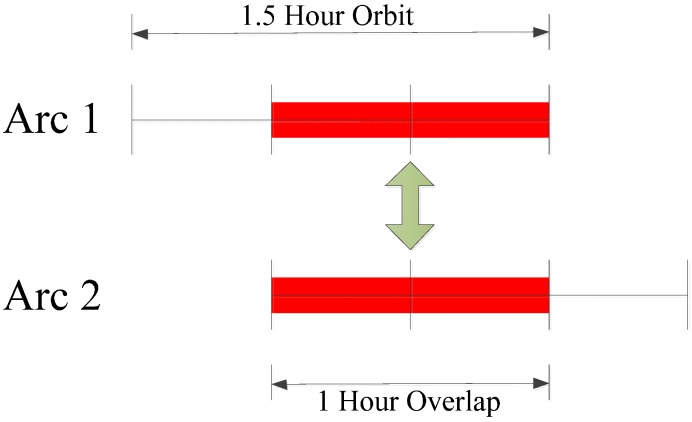
Orbit overlap comparison strategy.

**Figure 11:**
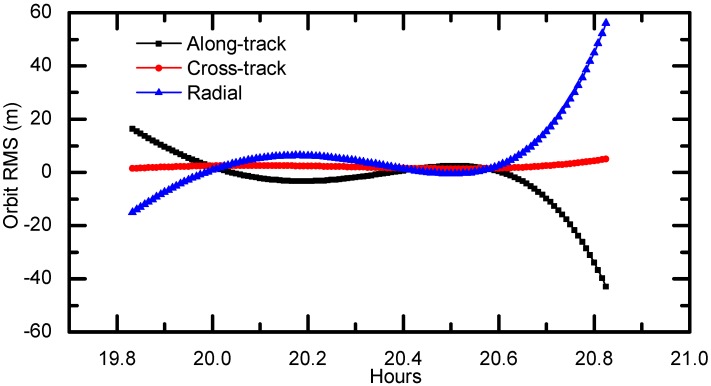
Orbit overlap comparison in radial, cross-track and along-track directions (unit: meter).

To evaluate dynamic orbit, we first separate the 2-h GPS data into two 1.5-h arcs with a 1-h overlap, as shown in Figure 10. These two arcs are processed with the same OD strategy presented in Section 3.1, and their orbit differences during the overlaps are calculated in along-track, cross-track and radial components and are used as indicators of the orbit quality. The overlap comparison here can reveal the internal consistency of the orbit estimates. Figure 11 shows the 1-h overlap differences. The differences in along-track and radial components are mainly within ±20 m but show larger discrepancies in the last 10 min. Comparatively, the orbit differences in cross-track component are much smaller. The RMS errors in along-track, cross-track and radial direction are 10.60 m, 2.26 m and 13.89 m, respectively. This indicates that the orbit consistency using the OD scheme presented in Table 2 should be at this precision.

### 3.3. Kinematic and Dynamic Orbit Comparison

There are typically two methods for GNSS-based spacecraft OD, i.e., dynamic and kinematic method. The dynamic method can generally obtain better orbit accuracy due to the constraint of dynamic models. While the kinematic method estimates the satellite’s position coordinates epoch-by-epoch and requires no a priori knowledge of the spacecraft motion [37]. The kinematic approach can be applied to a wide range of situations and is of particular interest for maneuvering spacecraft and reentry vehicles due to its purely geometrical nature. Moreover, the computational complexity is significantly reduced compared to the dynamical filtering technique.

**Figure 12:**
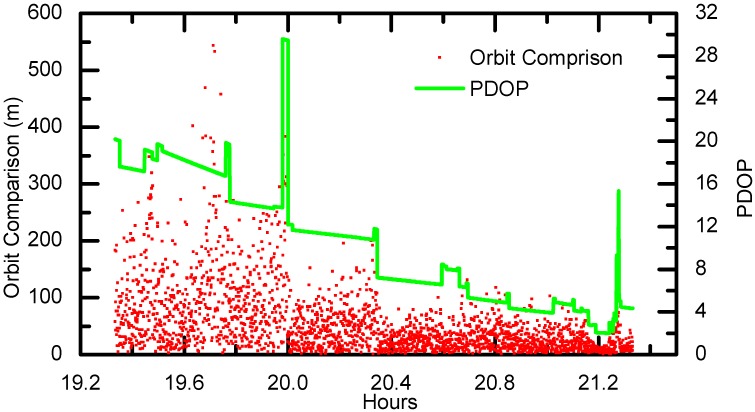
The SPP 3-Dimension (3D) precision and PDOP series.

The kinematic orbit of Chang’E-5T is also calculated in this study using the C1 code ranges by SPP approach. The differences between the kinematic orbits and the dynamic orbits can also reveal the quality of the orbit solutions. Ideally, the dynamic orbits are considered of higher accuracy as the carrier-phase observations are used in addition to the code ranges. Their differences are depicted in Figure 12. As shown, the differences show significant correlations with respect to the PDOP values, which is dominated by the spacecraft altitude. The kinematic orbit precision is roughly at 90 m level on average and can reach 50 m when the altitude reaches 20,000 km. This precision is consistent with the results from [28], which were obtained by OD using C1 code ranges combined with ground-tracking measurements.

### 3.4. Orbit Determination with Different C/N0 Thresholds

**Figure 13:**
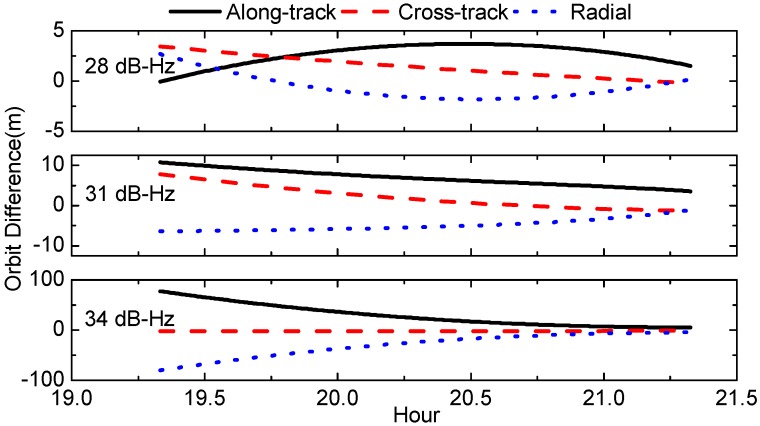
Orbit comparison solutions with different C/N0 thresholds in radial, cross-track and along-track directions (unit: meter).

In this section, we investigate the Chang’E-5T OD precision with different C/N0 thresholds, and the resultant orbits are compared with dynamic orbits. The orbit differences in along-track, cross-track, radial components are shown in Figure 13 and their RMS are listed in Table 3. 

It can be indicated that with lower C/N0 threshold, better orbit precision can be obtained. This can be primarily attributed to the number of visible GPS satellites decrement due to C/N0 threshold increment. When the C/N0 threshold is raised to 34 dB-Hz, the number of visible GPS satellites per epoch is around 2 as indicated in Section 2.1. This results in particularly low observation redundancy, and makes it more difficult for estimation convergence. Hence, for this case the orbit errors are as large as 80 m at the beginning and then converge slowly to smaller values; the overall 3D RMS errors are 50 m. However, for the other two cases, their orbit differences are both below 10 m in 3D RMS.

## 4. Conclusions

The 2-h onboard GPS data collected by Chang’E-5T probe are explored and analyzed in this contribution. The observation quality, mainly including the number of observed satellite, C/N0 level, PDOP as well as C1 code errors are evaluated in detail. It is found that on average the onboard GNSS receiver can track 7–8 GPS satellites per epoch for Chang’E-5T. For over 71.6% of the observations, their C/N0 values are higher than 31 dB-Hz, and for 31.8% over 34 dB-Hz. The PDOP values are significantly related to the spacecraft altitude, reaching about 20 at 50,000 km altitude and better than 10 below 30,000 km. Since the C1 and L1 observations are almost free from the ionosphere and troposphere delays, the C1 code error is calculated by differencing the C1 and L1 observations directly while the L1 ambiguities is removed by averaging. The resultant overall C1 code errors are 4.15 m. For C1 observations with higher C/N0 levels, i.e., 36 dB-Hz, the precision can be better than 2 m. Although increased C/N0 threshold can improve overall data quality and produce smaller code errors, the available number of GPS observation is greatly reduced and the resulting PDOP values are increased.

OD is carried out for Chang’E-5T using the C1 and L1 observations and the Helmert variance component estimation method is investigated to determine the weights of code and carrier phase observations. The OD precision is firstly evaluated by overlap comparison, which indicates an orbit consistency is about 17 m in 3D RMS. The RMS of L1 and C1 residuals are 74 mm and 4.58 m, respectively, showing good consistency with the C1 code errors statistics. Furthermore, the OD precision is analyzed by screening GPS data with different C/N0 thresholds of 28 dB-Hz, 31 dB-Hz and 34 dB-Hz. The results indicate that the OD precision for Chang’E-5T is mainly dominated by the number of visible GPS satellites. For the cases of C/N0 thresholds of 28 dB-Hz and 31 dB-Hz, the orbit differences are below 10 m. However, higher C/N0 threshold of 34 dB-Hz results in significant orbit precision degradation to 51 m, which should be primarily due to data volume decrement. Therefore, we suggest that the received C/N0 minimum should be designed to be less than 31 dB-Hz for onboard receiver in SSV, preferably less than 28 dB-Hz.

We need to further research in some aspects to improve the accuracy and autonomy of navigation architectures in future lunar exploration missions for the various mission phases. First, comprehensive utilization of GPS, GLONASS, BeiDou and Galileo system should be taken into account to improve OD accuracy by increasing the number of available satellites and reducing PDOP values. In addition, the integration of GNSS with other state-of-the-art space navigation sensors like IMU and Doppler radar altimeter is expected to achieve a high degree of autonomy and robustness of navigation. Second, a receiver with higher performance, particularly the capability of receiving weak signals, should be developed to provide GNSS navigation to the lunar explorers at the distance of 400,000 km and even farther in deep space. Third, the data processing algorithm should be adopted to further improve the accuracy and autonomy of navigation solutions using existing and future GNSS signals, such as real-time enhanced filtering and weak signal and low C/N0 data processing algorithms.

## Figures and Tables

**Table 1:** Primary parameters of the Chang’E-5T space-borne GNSS receiver.

Parameters	Design Parameter
Compatible frequency	GPS L1 and GLONASS L1
Original observation types	carrier phase, pseudo-range, Doppler and C/N0
Number of channels	16 for GPS, 8 for GLONASS
Sampling rate	3 s for ascent arc, 1 s for return arc
Acquisition sensitivity threshold	29 dB-Hz
Tracking sensitivity threshold	26 dB-Hz
GNSS Antenna	2, quadrifilar helix, earth-pointing and the opposite direction

**Table 2:** Observation models, dynamic models and estimated parameters for Chang’E-5T OD.

Items	Models
Estimator	Least-squares estimation
Observations selection	GPS L1 and C1
Sampling rate	3 s
Phase-windup effect	Phase polarization effects applied
GPS Satellite antenna phase center model (PCO and PCV)	Corrected using GPS values
GPS Satellite orbit	Fixed in IGS final orbit
GPS Satellite clock	Fixed in IGS final 30 s interval clock
Chang’E-5T Receiver clock	Estimated as random walk process
Precession and nutation	IAU 2000 precession and nutation model
EOP parameters	Polar motions and UT1 from IERS C04 series aligned to ITRF 2008
Troposphere and Ionosphere	None
Geopotential (static)	EIGEN_GL04C up to 50 × 50
Solid earth tide/Ocean tide/Solid earth pole tide/Relativistic effect	IERS Conventions 2003 [35]
M-body gravity	Sun, Moon, Jupiter, Venus, Mars, Mercury, Uranus, Neptune, Saturn, Pluto. JPL DE405 ephemeris used
Solar radiation pressure model	ECOM model 5-parameter with no initial value [36]
Phase ambiguities	Real constant value for each ambiguity arc

**Table 3:** Orbit Precision of different C/N0 thresholds (unit: meter).

C/N0	Along-Track	Cross-Track	Radial	3D
28	2.86	1.77	1.34	3.62
31	7.15	3.35	5.08	9.39
34	36.03	2.21	36.93	51.65
